# Protective Effects of Cannabidiol on Lesion-Induced Intervertebral Disc Degeneration

**DOI:** 10.1371/journal.pone.0113161

**Published:** 2014-12-17

**Authors:** João W. Silveira, Ana Carolina Issy, Vitor A. Castania, Carlos E. G. Salmon, Marcello H. Nogueira-Barbosa, Francisco S. Guimarães, Helton L. A. Defino, Elaine Del Bel

**Affiliations:** 1 Department of Morphology, Physiology and Basic Pathology, Dental School, University of São Paulo (USP), NAPNA, Ribeirão Preto, Brazil; 2 Department of Physics, Philosophy and Sciences School, University of São Paulo (USP), Ribeirão Preto, Brazil; 3 Division of Radiology, Medical School, University of São Paulo (USP), Ribeirão Preto, Brazil; 4 Department of Pharmacology, Medical School, University of São Paulo (USP), NAPNA, Ribeirão Preto, Brazil; 5 Department of Biomechanics, Medicine and Rehabilitation of the Locomotor Apparatus, Medical School, University of São Paulo (USP), Ribeirão Preto, Brazil; National Institutes of Health, United States of America

## Abstract

Disc degeneration is a multifactorial process that involves hypoxia, inflammation, neoinnervation, accelerated catabolism, and reduction in water and glycosaminoglycan content. Cannabidiol is the main non-psychotropic component of the Cannabis sativa with protective and anti-inflammatory properties. However, possible therapeutic effects of cannabidiol on intervertebral disc degeneration have not been investigated yet. The present study investigated the effects of cannabidiol intradiscal injection in the coccygeal intervertebral disc degeneration induced by the needle puncture model using magnetic resonance imaging (MRI) and histological analyses. Disc injury was induced in the tail of male *Wistar* rats via a single needle puncture. The discs selected for injury were punctured percutaneously using a 21-gauge needle. MRI and histological evaluation were employed to assess the results. The effects of intradiscal injection of cannabidiol (30, 60 or 120 nmol) injected immediately after lesion were analyzed acutely (2 days) by MRI. The experimental group that received cannabidiol 120 nmol was resubmitted to MRI examination and then to histological analyses 15 days after lesion/cannabidiol injection. The needle puncture produced a significant disc injury detected both by MRI and histological analyses. Cannabidiol significantly attenuated the effects of disc injury induced by the needle puncture. Considering that cannabidiol presents an extremely safe profile and is currently being used clinically, these results suggest that this compound could be useful in the treatment of intervertebral disc degeneration.

## Introduction

Intervertebral disc (IVD) degeneration is believed to be the main contributor agent for chronic low back pain. It is a major public health problem with great socioeconomic impact worldwide. IVD degeneration is a multifactorial process characterized by serial progressive changes in the morphology, biochemical components and biomechanical function of the IVD [1], [2].

The IVD is identified as an immune-privileged organ with no access to systemic circulation [3]. However, several pieces of evidence support the prominence of the inflammatory response in the pathogenesis of IVD degeneration [4], [5], [6]. In fact, there is an up-regulation of inflammatory factors that shift homeostasis of the extracellular matrix towards a degenerative and catabolic state, with subsequent breakdown of its components [7], [8]. On the other hand, there is conflicting information regarding the efficacy of intradiscal steroid injections in the treatment of IVD [9], [10], [11], [12]. A clinical study conducted by Buttermman (2004) [13] suggested that intradiscal steroid injections are beneficial for a small number of patients with advanced IVD degeneration. This therapeutic strategy could be more effective in special circumstances in which patients have inflammatory end-plate alterations classified as Modic Type I changes [13], [14].

Cannabidiol (CBD) is the major nonpsychotropic phytocannabinoid of *Cannabis sativa* (up to 40% of *Cannabis* extracts). Contrary to most cannabinoids, CBD does not produce psychotomimetic or cognitive effects [15], [16]. Interesting, in the last years it has been suggest that CBD produces a plethora of others pharmacological effects, including antioxidant [17], neuroprotective [18], [19], [20], [21], [22], anti-proliferative [23], [24], anti-anxiety [25], [26], hypnotic and antiepileptic [27], anti-nausea [28], anti-ischemic [29], anti-hyperalgesic [30], and anti-inflammatory [31], [32], [33]. In humans, CBD has been tested in preliminary trials related to diseases such as rheumatoid arthritis [34], [35], multiple sclerosis [36], [37], [38], anxiety [16] and psychosis [39], and shows an extremely safe profile [40], [41]. However, the effects of CBD on the treatment of IVD degeneration have not been investigated yet.

The purpose of this study was to evaluate by magnetic resonance imaging (MRI) and histological analysis the effects of intradiscal injection of CBD in an induced coccygeal disc degeneration model.

## Material and Methods

### Animals

A total of 19 male *Wistar* rats (300–350 g) were used in this study. Animals were housed in groups of 4, and kept at a temperature of 23±1°C with a 12 hours light-dark cycle. Food and drinking water were available *ad libitum*. All experiments were conducted according to the principles and procedures described by the Guidelines for the Care and Use of Mammals in Neuroscience and Behavioural Research (Institute for Laboratory Animal Research, USA) and the Guidelines of the School of Medicine (USP, Brazil), whose Animal Ethics Committee for Animal Experimentation analysed and approved the experimental procedures (protocol number 016/2012).

### IVD lesion

The animals were anesthetized with ketamine (50 mg/kg) and xylazine (5 mg/kg), injected intraperitoneally (i.p). The coccygeal intervertebral levels Co6–7, Co7–8, Co8–9 and Co9–10 were selected for the study. The details of the experimental design and the induction of IVD lesion by needle puncture in the tail of *Wistar* rats have been recently published [42]. This methodology was used here with small modifications. Briefly, two non-contiguous discs Co6–7 and Co8–9 were used to induce disc degeneration by needle puncture. The IVD Co7–8 and Co9–10 remained undisturbed to be used as control levels. The IVD Co9–10 received vehicle injection with a 30-gauge needle. The 21-gauge needle was inserted into Co6–7 and Co8–9 at the level of the annulus fibrosus (AF), crossing the nucleus pulposus (NP) up to the contralateral AF. After full penetration, the needle was rotated 360° twice and held for 30 sec.

### Intradiscal Injection of CBD or vehicle

**Figure 1:**
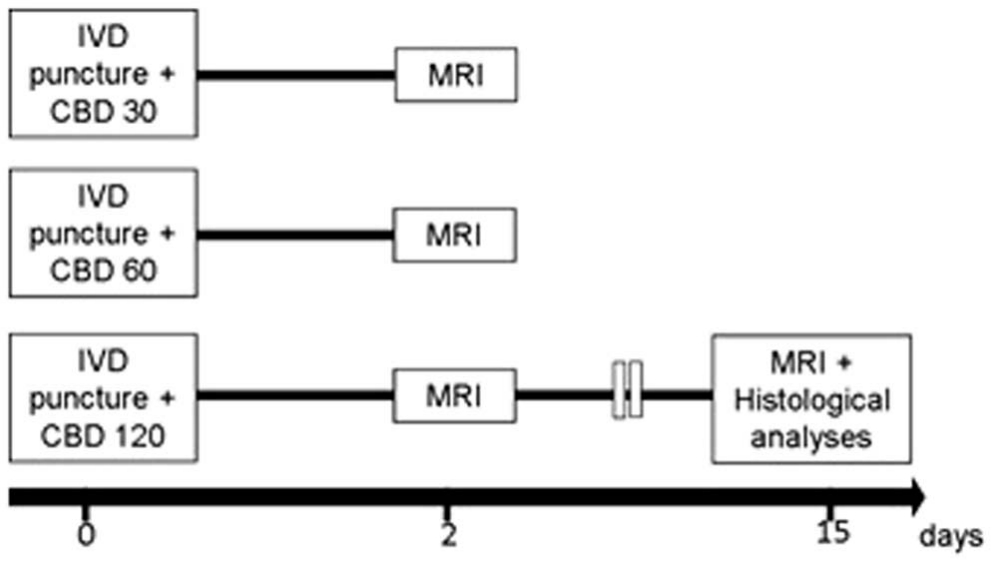
Schematic time line representation of the experimental design. All animals were submitted to MRI two days after the IVD puncture and CBD (30, 60 or 120 nmol) microinjection. Animals that received CBD 120 nmol microinjection were resubmitted to MRI at the 15^th^ day post-lesion and then to the histological analyses.

The animals were divided into three sub-groups (with 6–7 animals each) according to CBD doses (Fig. 1). Under general anesthesia, immediately after the disc puncture, 2 µl of CBD (30, 60 or 120 nmol) diluted in 98% saline and 2% Tweem-80 was injected into the Co6–7 lesioned discs. Likewise, the lesioned discs Co8–9 and the non-lesioned discs Co9–10 were injected with vehicle (saline + Tweem-80; 2 µl). Both CBD and vehicle injections were conducted using a Hamilton micro-syringe attached to a 30-gauge needle.

### MRI acquisition

The MRI was performed under general anesthesia 2 and 15 days after the IVD puncture. Images were acquired in a 3.0 T MR scanner (Philips, Achieva, The Netherlands) using a dedicated coil for small animal. The tail was inserted into a tub containing a 0.1 M CuSO_4_ solution to increase the contrast in the image. A 2-D spin- echo, dual echo sequence was obtained with the following parameters: repetition time  = 9000 ms, echo times  = 16 (Proton density, PD) and 80 (T2-weigthed) ms, flip angle  = 90, numbers of averages = 2, slice thickness = 0,6 mm, field of view  = 40×40 mm, in plane resolution  = 0.1 mm, 30 sagittal slices. A linear combination of both images (PD and T2) showed excellent anatomical details and it was used to perform a qualitative evaluation of disc integrity. The disc signal intensity was estimated in the T2-weighted image (Echo time  = 80 ms) as an indirect measure of disc conditions and its water content. Four discs from Co6–7 to Co9–10 for each animal were analyzed using the Image J software. In order to evaluate the entire disc, five sequential sagittal images were considered and the mean pixel intensity value was quantified.

### Histological score

Animals were sacrificed by CO_2_ inhalation 15 days after the disc puncture. IVDs were removed, fixed in buffered 4% PFA for 24 hours and then subjected to the decalcification process in an ETDA solution consisting of 12% hydrochloric acid, 0.07% EDTA, 0.014% sodium tartrate, 0.8% sodium and potassium tartrate, in water, during 24 hours. After descaling, IVDs were dehydrated, embedded in paraffin and 5 µm sections were obtained with a microtome (Leica RM2145). The sections were then stained with hematoxylin and eosin for histological score and graded by an observer blind to the treatment conditions using the definition established by Norcross et al. 2003 [43], with some modifications (Table 1), under a light microscope (Leica, Germany) at 5× magnification. A grade score ranged from 1 (several degenerated disc) to 5 (normal disc) was assigned separately to both AF and NP.

**Table 1:** Histologic grading scale.*

Nucleus pulposus (NP)
**5** Large, bulging central cavity with abundant NP material;>2/3 IVD height; smooth borders with minimal disruption;
**4** Slightly reduced central cavity size with some NP material present;>1/3 IVD height and <2/3 IVD height; minimal border disruption may be present;
**3** Markedly reduced and disrupted cavity with minimal NP material and compartmentalization; total cavity>1/3 IVD height and <2/3 IVD height;
**2** Severe disruption of NP with minimal cavity; total cavity <1/3 IVD height but>0; consists only of a few small pockets lined by NP-like cells;
**1** Complete obliteration of cavity with no NP-lined pockets.

Annulus fibrosus (AF)
**5** Discrete, well-opposed lamellae bulging outward with no infolding; minimal preparation defect with “simple radial clefting”;
**4** Discrete lamellae, less well-opposed; minimal infolding may be present; fibers remain well-organized, but with “complex radial clefting”;
**3** Moderate to severe infolding of discrete, relatively well-opposed lamellae; moderate fragmentation of lamellae; AF fibers remain well organized;
**2** Severe infolding and distortion of poorly opposed lamellae; severe fragmentation of lamellae; small regions of disorganized fibrous material replacing central lamellae;
**1** Severe infolding, distortion, and fragmentation of lamellae; extensive amount of disorganized fibrous material replacing central lamellae.

*This scale mainly scores the disruption of nucleus pulposus central cavity and cellularity, and collagen fiber orientation of annulus fibrosus. Simple radial clefting  =  the presence of radial gaps between AF lamellae with minimal fragmentation; complex radial clefting  =  presence of radial, transverse, and/or oblique gaps in the lamellae with significant fragmentation. Based on Norcross et al., 2003.

### Statistical Analysis

The MRI data (mean ±SEM; n = 5–7) was analyzed by one-way ANOVA followed by the Newman-Keuls Multiple Comparison Test. The histological scores were analyzed by the non-parametric Kruskal-Wallis test followed by Mann-Whitney and data was represented as the median value. In all cases, the level of significance was set at p<0.05.

## Results

### Experimental validation: effectiveness of the puncture lesion model

**Figure 2:**
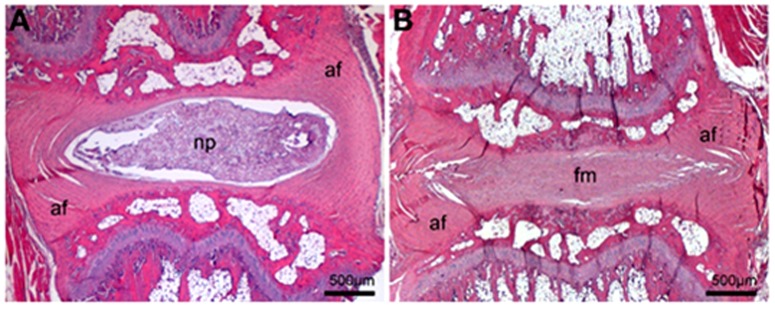
Representative histological sections of IVD. (A) Intact disc showing well-organized fibers in the annulus fibrosus (af) with clearly defined border, and nucleus pulposus (np) comprising a significant disc area, with minimal border disruption. (B) IVD 15 days after lesion and vehicle injection showing an AF with lamellar disorganization, cavity obliteration with no NP cells and height decrease. (fm) fibrous material; sagittal sections; hematoxylin and eosin, 5X.

Fig. 2 shows the typical morphology of the intact IVD, which presents an AF with well-organized fibers and a clearly defined border. The NP comprises a significant disc area in the sagittal sections with minimal disruption in its border. Clear histological changes can be observed in the IVD after lesion with the needle puncture. Disc sections showed an AF with lamellar disorganization/fragmentation. NP presented complete obliteration of its cavity with fibrous material. Also, there is a significant decrease of the IVD height.

**Figure 3:**
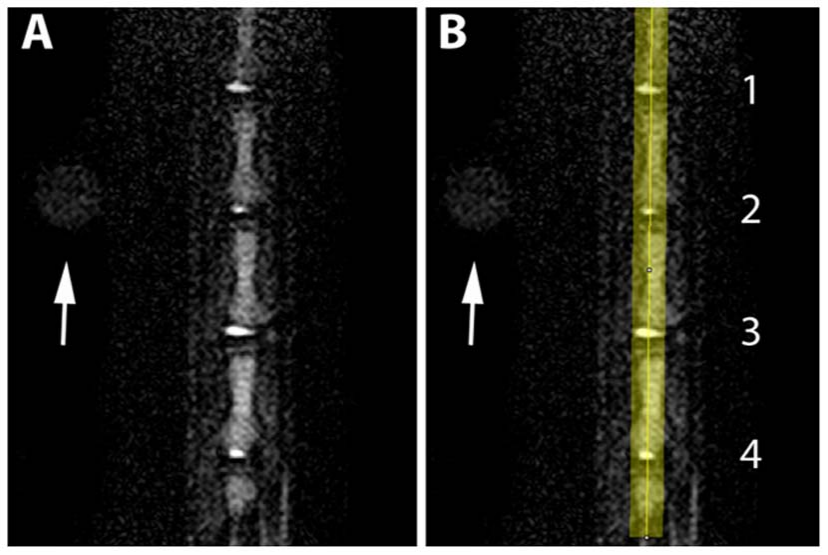
Representative serial T2-weighted midsagittal MRI. **A** and **B**, T2-weighted MRI obtained in four rat tail IVDs. The arrow indicates a reference body used to localize the Co7–8 disc in the image. **1** Co9–10 intact: vehicle injection; **2** Co8–9 21-gauge needle puncture: vehicle injection; **3** Co7–8 intact; **4** Co6–7 21-gauge needle puncture: CBD injection.

The MR images of the NP in the punctured discs showed weaker signal intensities than those of the intact control discs two days after needle puncture. In the NP, decreased water, proteoglycans and increased collagen content are classical technical features of degenerated IVD, which can be visualized on MRI with T2 weighting as a hypointense signal. Representative serial T2-weighted midsagittal images of the intact and punctured tail discs are shown in Fig. 3.

### Effects of intradiscal injection of cannabidiol (30, 60 or 120 nmol)

**Figure 4:**
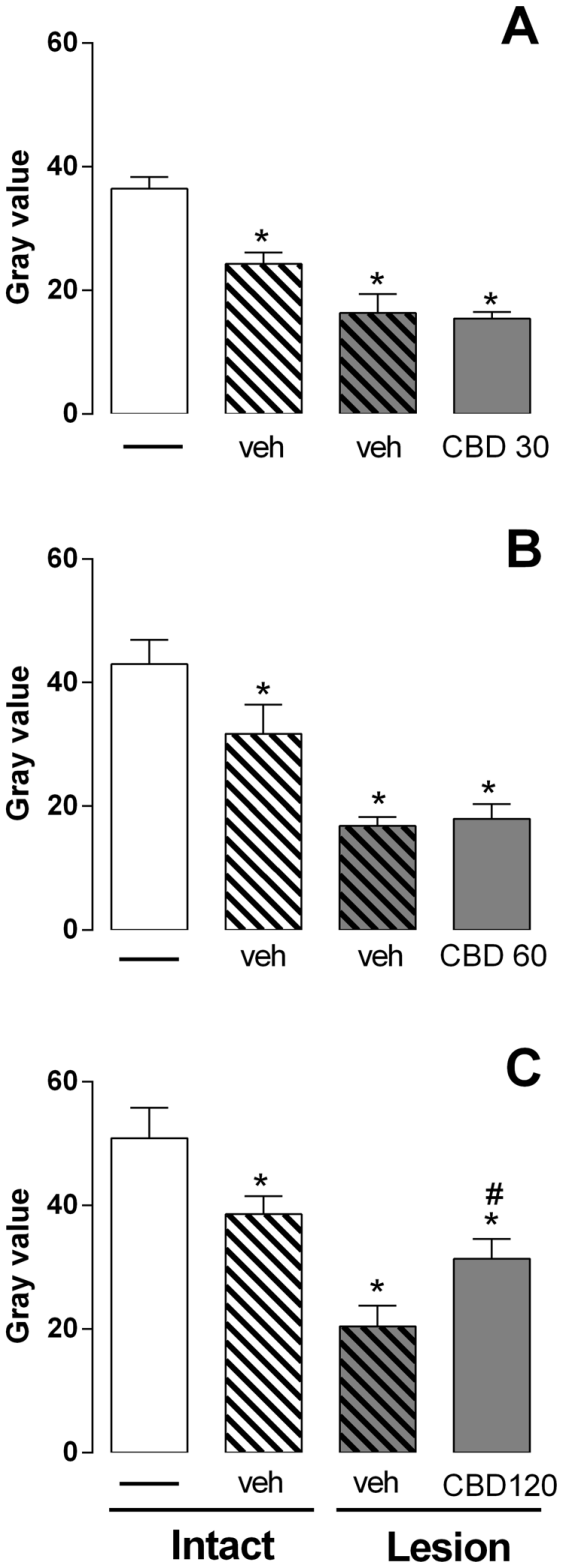
MRI evaluation 2 days after lesion and CBD injection. The injection of CBD 30 (A) or 60 nmol (B) did not prevent the MRI signal reduction induced by needle puncture. CBD 120 nmol injected into the punctured discs mitigates the MRI changes (C). The injection of vehicle *per se* caused significant changes in the MRI signal intensity of the non-puncture discs compared to the intact discs (A–C). *p<0.05 *versus* intact discs. # p<0.05 *versus* vehicle injected lesioned discs.

IVD lesion caused significant reduction in the MRI signal intensity two days after the needle puncture. The punctured discs showed a significant decrease in MRI pixel intensity two days after the procedure compared to the intact discs that received or not vehicle injection Co9–10/Co7–8, respectively (Fig. 4 A: [F_(3,20)_ = 27.58, p = 0.0001]; B: [F_(3,17)_ = 9.233, p = 0.0008] and C [F_(3,20)_ = 12.07, p<0.0001]). Vehicle injection *per se* in the intact discs caused a significant reduction in MRI pixel intensity compared to the non-injected discs in all experimental groups (Fig. 4).

Treatment with CBD 30 or 60 nmol immediately after lesion did not modify MRI pixel intensity (Fig. 4A and B). Microinjection of CBD 120 nmol immediately after lesion significantly improved MRI pixel intensity two days after the injection compared to the vehicle injected lesioned discs (Fig. 4C).

### Long-term effects of intradiscal injection of cannabidiol (120 nmol)

**Figure 5:**
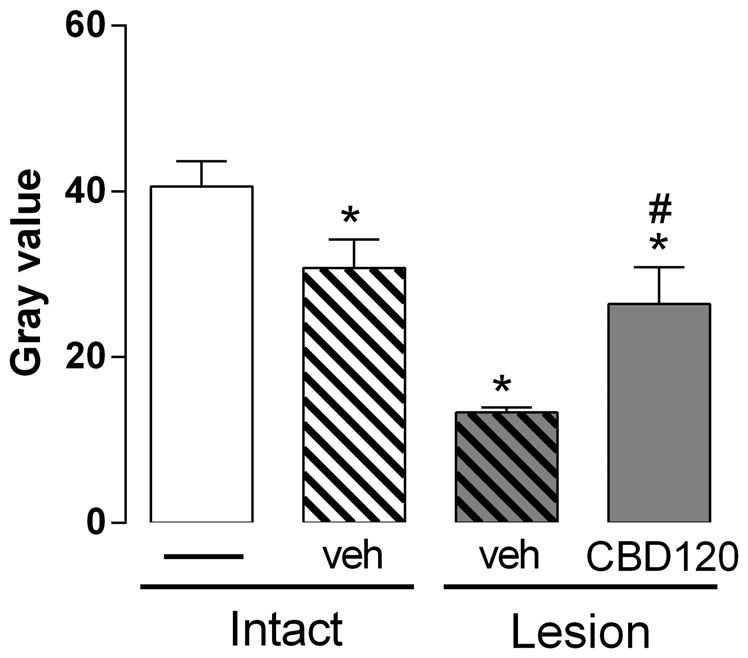
MRI evaluation 15 days after lesion and CBD injection. The injection of CBD 120 nmol into the punctured discs mitigates the MRI changes. The injection of vehicle *per se* caused significant changes in the MRI signal intensity of the non-puncture discs compared to the intact discs. *p<0.05 *versus* intact discs. # p<0.05 *versus* vehicle injected lesioned discs.

Fifteen days after the puncture procedure discs which received vehicle injection (Co8–9) showed a significant decrease in MRI pixel intensity compared to the intact disc that received or not a vehicle injection (Co9–10/Co7–8, respectively; [F_(3,19)_ = 14.10, p<0.0001]; Fig. 5).

Injection of CBD 120 nmol immediately after lesion significantly improved MRI pixel intensity. The effect was maintained at least for 15 days after injection compared to the vehicle injected lesioned discs (Fig. 5).

**Figure 6:**
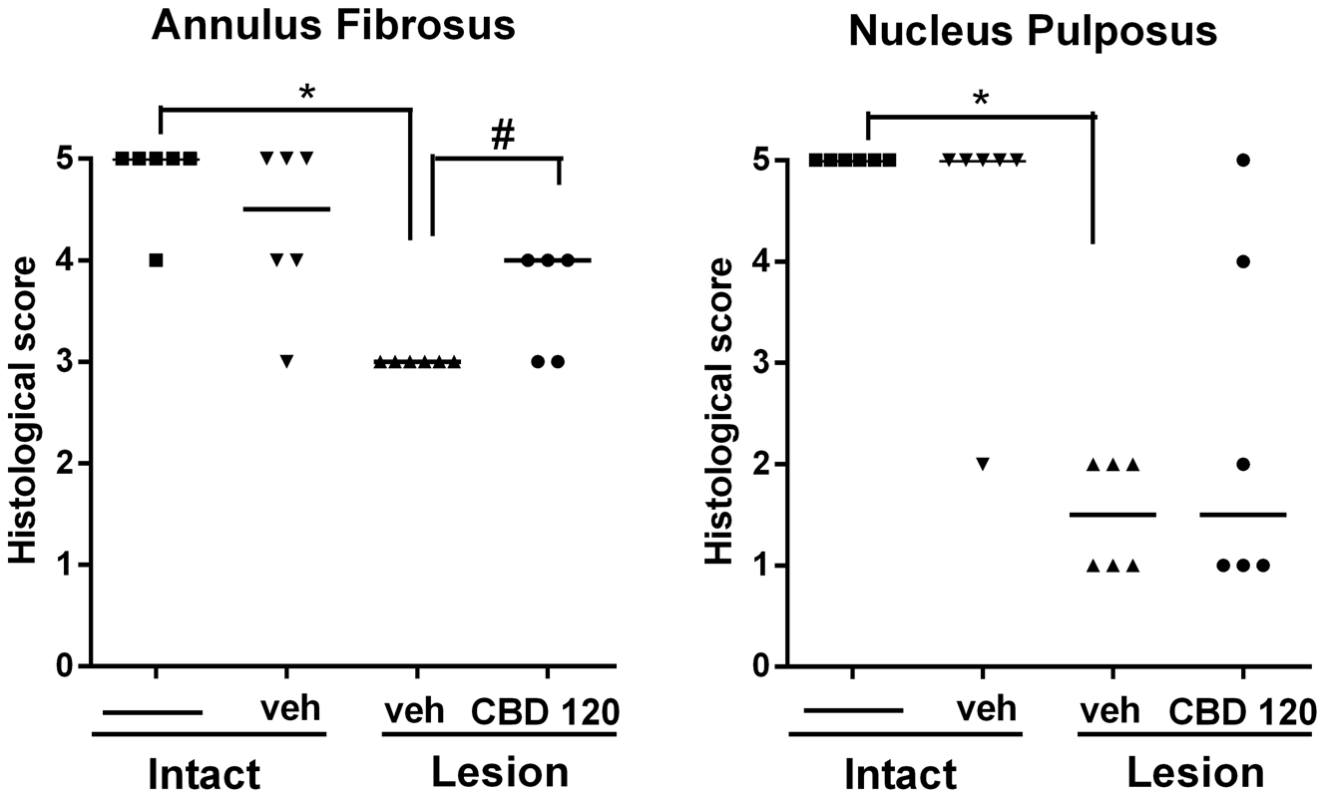
Effects of CBD on histological scores 15 days after IVD puncture. IVDs that received an injection of vehicle after needle puncture showed significant worse histological scores. CBD 120 nmol injection significantly improved histological scores of the AF. *p<0.05 *versus* intact discs. # p<0.05 *versus* vehicle injected lesioned discs.

MRI data was corroborated by histological results. The histological score was evaluated at fifteen days after the lesion and injection of vehicle or CBD 120. The lesion decreased the histological scores in the AF and NP. CBD 120 nmol prevented the typical histological changes in the AF. No histological changes were observed when the vehicle was injected into the intact disc (Fig. 6).

## Discussion

The primary objective of this study was to investigate the ability of CBD to decrease degenerative events induced by needle puncture of the IVD. Three concentrations of CBD were injected into the IVDs immediately after the disc puncture.

The beneficial effect of CBD, as measured by high resolution MRI, was evaluated two days after the injection in all experimental groups. Only with the concentration of 120 nmol an improvement in the MRI signal intensity was observed. Following this result, an additional analysis was conducted 15 days after CBD 120 nmol injection.

MRI is a non-invasive technique that is the most important method for clinical assessment of IVD pathology. The signal loss on T2-weighted MRIs correlates with the progressive degenerative changes of the IVD [44], [45]. The brightness of the NP has been shown to directly correlate with proteoglycan concentration [46], even more than gross tissue morphology [47]. Higher proteoglycan content in the NP induces water accumulation and increases MRI signal. This region is most severely affected during disc degeneration and, consequently, is a focus for novel cell-based regenerative strategies [48]. However, the 30-gauge needle used for vehicle injection induced significant changes in MRI pixel intensity of the non-puncture discs. Therefore this methodology must be used with considerable caution.

Histological features of the degenerating IVD include specific changes of the NP and AF [43], [49], [50]. The NP shows severe disruption and a gradual decrease in cell number, with reduction in its cavity until complete obliteration. This is consistent with the loss of proteoglycan seen in degenerative models [43], [49], [50] and in human degenerated discs [51]. The AF had radial gaps between lamellae with fragmentation culminating in regions of disorganized fibrous material replacing central lamellae [43], [49], [50]. CBD treatment was able to attenuate the decrease in histological scores induced by the lesion in the AF, but not in the NP region. This lack of correlation between the MRI and histological analysis in the latter region suggest, at least in the present model, a higher sensitivity of the former method.

Multiple mechanisms of action have been proposed to explain CBD effects. It could antagonize the cannabinoids receptors CB1 and CB2 in low nanomolar range and to function as an inverse agonist at 1–10 µM [52]. However, to date, the presence of cannabinoids receptors has not been described in the IVD. CBD also acts as an inhibitor of anandamide uptake and hydrolysis enzymes [53], which results in enhanced levels of this endocannabinoid that might account for the anti-hyperalgesic and anti-inflammatory actions of CBD. Moreover, as a vanilloid receptor agonist with potency equivalent to capsaicin, CBD can desensitize vanilloid receptor 1 and leads to analgesic and anti-inflammatory effects [53]. The competitive inhibition of adenosine uptake and the resulting enhancement of adenosine signaling can also be involved in the anti-inflammatory effects of CBD [54].

Considering that the modulation of immune responses in the degenerate disc is essential for the recovery of this immune-privileged organ [3], [55], the effects of CBD in lesion-induced degeneration could depend on its anti-inflammatory profile. Disc degeneration involves the release of many inflammatory signaling molecules [31], [56], [4], [5], [6]. In most models of inflammation, CBD attenuates inflammatory cell migration/infiltration [57], [33] and the production of inflammatory mediators [58]. For example, CBD suppresses proinflammatory signaling, including NF-κB, induced by LPS [56]. Time- and dose-dependent anti-inflammatory activity has also been observed in acute inflammation models [31]. CBD is also effective in chronic neuropathic painful states that are associated with the release of proinflammatory cytokines, such as IL-6, IL-1β, and TNFα [20], [33], [56]. Moreover, CBD can abolish the increase of nitric oxide levels in paw tissues in inflammatory and neuropathic pain models [59]. Finally, the ability of CBD to reduce inflammatory markers has been shown in several experimental different conditions. CBD treatment inhibits the progression of periodontitis that was accompanied by a decrease in neutrophil migration, expression of bone related molecules and production of IL-1β and TNF-α [33]. In arthritis models, CBD blocks its progression, decreasing collagen II-specific proliferation and IFN-γ production, as well as decreasing the release of TNF-α by synovial cells [35]. In diabetic retina, the increase in phosphorylation of p38 MAP kinase, a stress-activated protein kinase that is a downstream target of proinflammatory cytokines, is blocked by CBD treatment [60]. Additionally, CBD beneficial effects have been recently described in the liver injury model of hepatic ischemia/reperfusion by attenuating inflammatory signaling in a CB1/2 receptors independent manner [61], [62].

In summary our study revealed anti-degenerative effects of intradiscal microinjection of CBD 120 nmol. CBD represents one of the most promising candidates present in the *Cannabis sativa* plant for clinical use due to its remarkable lack of cognitive or psychotomimetic actions. It has been already approved in several countries for the treatment of neuropathic pain [63]. Although further research is necessary to clarify the mechanisms involved in CBD effects, the present results suggest the possibility of its use for disc degeneration treatment.
